# Early recurrence of febrile seizures during acute illness: risk factors and lack of association with long-term epilepsy in a Pediatric cohort

**DOI:** 10.3389/fneur.2025.1733941

**Published:** 2026-01-06

**Authors:** Weiqin Jiang, Anna Cheng, Jing Wang, Renjian Wang, Shan Zhang, Yujuan Huang

**Affiliations:** Department of Emergency, Shanghai Children’s Hospital, School of Medicine, Shanghai Jiao Tong University, Shanghai, China

**Keywords:** acute-phase recurrence, febrile seizures, influenza A, long-term recurrence, risk factors

## Abstract

**Background:**

Febrile seizures (FSs) are a common neurological manifestation in children, affecting 2%–5% of children worldwide. While acute-phase recurrences during the same febrile illness are frequent, their risk factors and implications for long-term epilepsy remain incompletely understood. This study aimed to identify independent risk factors for recurrent FSs (RFS) during the acute febrile phase and clarify their association with long-term epilepsy development.

**Materials and methods:**

This retrospective cohort study included 611 children (aged 6 months to 3 years) diagnosed with FSs at a tertiary pediatric hospital in Shanghai between April 2021 and March 2023. Clinical data on seizure recurrence patterns, demographic characteristics, and influenza A infection status were collected. Multivariate logistic regression was used to identify independent risk factors for acute-phase RFS. Long-term outcomes, including subsequent seizure recurrence and epilepsy incidence, were compared between children with and without RFS using Kaplan–Meier survival analysis, with differences assessed by the log-rank test.

**Results:**

The median time to recurrence was 6 h after the initial seizure, and 94.95% of recurrent events occurred within 24 h. Independent risk factors for acute-phase RFS were a prior history of FSs [odds ratio (OR) = 2.24, 95% confidence interval (CI): 1.42–3.56] and positive influenza A infection (OR = 3.34, 95% CI:1.96–5.71). Among 99 children with RFS, 22.22% experienced further seizures and 2.02% developed epilepsy during a median follow-up of 39 months (interquartile range: 37–41). In the non-recurrent FS group (*n* = 512), 19.14% had later seizures and 0.39% developed epilepsy. Although any subsequent seizure (febrile or afebrile) was more frequent in the RFS group, the difference in long-term seizure risk was not statistically significant [log-rank *χ*^2^ = 0.74, *p* = 0.391; hazard ratio (HR) = 1.223, 95% CI: 0.770–1.942].

**Conclusion:**

Acute-phase FSs recurrence is common and typically occurs within 24 h, with prior FSs history and influenza A infection as key risk factors. However, early recurrence was not associated with increased long-term epilepsy risk. These findings support close monitoring of high-risk children during febrile illnesses while alleviating unnecessary concerns regarding future neurologic outcomes.

## 1 Introduction

Febrile seizures (FSs) are a common neurological manifestation in children, affecting 2%–5% of children worldwide (1). They are defined as generalized or focal seizures occurring in association with fever in the absence of central nervous system infection, and incidence peaks between 6 months and 5 years of age (1–3). While the majority of FSs are self-limiting and carry a favorable prognosis, early recurrence during the same febrile illness poses significant challenges for clinicians and families. Recurrent seizures within 24 h occur in 14%–24% of cases (4–9), often necessitating emergency department visits, prolonged hospitalizations, and increased parental anxiety (10, 11). Beyond immediate management concerns, a critical knowledge gap persists regarding whether early FS recurrence independently influences long-term outcomes, particularly the risk of subsequent epilepsy.

Existing literature identifies several risk factors for FS recurrence, including low peak temperature at seizure onset (10, 12–14), male sex (10), positive family history of FS (15), focal seizures, and prolonged first seizure duration (4, 13). However, studies investigating the relationship between early FS recurrence and long-term epilepsy have yielded conflicting results. While some retrospective analyses suggest an association between multiple FSs and subsequent epilepsy (16, 17), others argue that early recurrence alone does not independently elevate epilepsy risk (18). This ambiguity stems, in part, from methodological limitations, including small sample sizes, variable definitions of “early recurrence,” and insufficient longitudinal follow-up. In addition, a recent multicentre study has delineated specific clinical red flags—such as meningeal signs, focal features, or prolonged duration—that not only help exclude life-threatening differential diagnoses but also predict subsequent FS recurrence and later epilepsy risk (19).

To address these gaps, we conducted a retrospective cohort study of 611 children presenting with FSs during a febrile illness. Leveraging detailed clinical data and three-year follow-up records, this study aims to: (1) identify independent risk factors for early FS recurrence (<24 h) during the acute illness; (2) clarify the association between early recurrence and the development of epilepsy; and (3) establish a predictive model to guide clinical decision-making. By integrating multivariate logistic regression and survival analysis, we provide robust evidence to refine risk stratification for FS recurrence and alleviate unnecessary concerns regarding long-term epilepsy risk in this vulnerable population.

## 2 Subjects and methods

### 2.1 Study design and population

This study was designed as a retrospective cohort analysis of children hospitalized for FS, with a prospective follow-up component to assess long-term outcomes. This investigation included 611 pediatric cases (aged 6 months to 3 years) who presented with fever-associated convulsive episodes at the emergency department of Shanghai Children’s Hospital from April 2021 to March 2023. All participants conformed to the modified 2011 American Academy of Pediatrics (AAP) criteria for FSs (3). Specifically, FSs were defined as follows: Convulsive episode(s) occurring in children with a core temperature of ≥38.5 °C (rectal) or ≥38 °C (axillary), and there was no clinical or imaging evidence of central nervous system infection, metabolic encephalopathy, previous afebrile seizures, cerebral trauma, or congenital malformations.

Exclusion criteria were as follows: pre-evaluation multiple seizure episodes; transferred patients who had received prior antiseizure medication administration; confirmed epilepsy diagnosis; neonatal seizure etiology; provoked seizures (toxic/metabolic/electrolyte imbalance); and insufficient medical records.

### 2.2 Operational definitions

Recurrent FS (RFS): Two or more discrete convulsive episodes within the acute febrile illness period. The illness episode began with the index FS and ended when the child remained afebrile (core temperature <37.5 °C) for ≥24 h (10).

Non-recurrent FS (NRFS): A single seizure occurrence during clinical follow-up.

Seizure recurrence was defined as any subsequent seizure event and therefore encompassed both (i) recurrent FS occurring during a future febrile illness and (ii) new-onset unprovoked seizures (epilepsy).

In accordance with the LICE Guidelines (1), each FS was classified as simple (generalized, duration <15 min, no recurrence within 24 h) or complex (focal, ≥15 min, or ≥2 seizures within 24 h). Owing to perfect collinearity (all acute-phase recurrences met complex criteria), the simple/complex variable was not entered into multivariable models.

### 2.3 Clinical management protocol

#### 2.3.1 Standardized acute-phase care comprised the following aspects

Positioning: Lateral decubitus with airway protection.

Oxygenation: Low-flow O_2_ (2–4 L/min) administered via nasal cannula.

#### 2.3.2 For the seizure termination algorithm

If there was spontaneous resolution within ≤ 3 min, supportive monitoring was carried out.

If there was persistent seizure activity, the first-line treatment was intramuscular midazolam (0.1–0.3 mg/kg). In refractory cases, intravenous diazepam (0.1–0.3 mg/kg) was administered.

### 2.4 Data collection framework

Demographic data, clinical characteristics of FSs, and results from laboratory tests and imaging studies were systematically extracted from the patients’ electronic health records. The collected data included gender, age, body temperature at the onset of FSs, time interval from fever onset to the occurrence of FSs, duration of FSs, prior personal history of FSs, family history of FS, results of influenza testing, electroencephalogram (EEG) findings, cerebrospinal fluid (CSF) analysis obtained through lumbar puncture, brain magnetic resonance imaging (MRI) results, and clinical outcomes.

Children presenting with their first seizure of the current febrile illness were admitted to the emergency department of our hospital within 24 h after the seizure event. The attending physician conducted a comprehensive medical history interview with the parents or guardians, recorded the child’s vital signs, and documented the specific characteristics of the seizure. All temperatures were measured with the same infrared tympanic thermometer (Braun ThermoScan 7) at enrolment and every subsequent follow-up visit, and are reported exclusively in °C. All children underwent influenza testing. Nasopharyngeal swabs were collected within 2 h of arrival and analysed by colloidal-gold antigen rapid test (20, 21) or by RT-PCR; only influenza A is included in the hospital’s winter febrile-seizure rapid-screen protocol. Disposition (inpatient vs. outpatient) was determined by the attending physician according to a comprehensive clinical assessment.

All participants were re-evaluated within 1 week to identify acute-phase recurrences, defined as new seizures with concomitant fever. Long-term follow-up continued until April 2025, with the primary outcome being any subsequent seizure. Time-to-event was calculated from study entry to the first verified recurrent seizure or the last follow-up date (April 2025), whichever occurred first, and expressed in months.

#### 2.4.1 Criteria for electroencephalography, magnetic resonance imaging, and lumbar puncture

Indications for ancillary investigations were prospectively defined in a standardized clinical checklist to minimize individual subjective bias. EEG was performed for focal or prolonged seizures (>10 min), any post-ictal neurological deficit, or recurrence within 24 h. Brain MRI was indicated in the presence of focal semiology, focal neurological signs on examination, or ≥ 2 complex features. Lumbar puncture was performed if any of the following were present: age < 12 months, meningeal signs, altered consciousness persisting >1 h, or fulfilment of clinical sepsis criteria. Children who met any criterion were counselled and offered the corresponding investigation unless parents declined; consequently, the uptake rate for each test reflected clinical presentation rather than investigator selection.

#### 2.4.2 Long-term verification protocol

Long-term verification protocol: participants were enrolled at discharge and prospectively followed via a three-tier surveillance system.

(1) Electronic medical records automatically flagged scheduled revisits and extracted any emergency or outpatient encounter coded for seizures (ICD-10 G40.- or R56.8).

(2) Structured telephone/WeChat interviews using a 12-item questionnaire were administered at 1, 6, 12, 24, and 36 months (±2 weeks); items captured date/time of event, core temperature (≥38 °C), seizure semiology (ictal topography, duration, symmetry), loss of consciousness, medical attendance, antiseizure-medication use, prior EEG/MRI, post-ictal neurodevelopment, availability of video documentation, discharge-summary acquisition, respondent relationship, and call duration. All interviews were audio-recorded; 10% were randomly selected and independently reviewed for quality control.

(3) Any parent-reported episode suggestive of an unprovoked or complex seizure prompted an outpatient evaluation within 7 days, during which a paediatric neurologist corroborated the history, performed a neurological examination, and ordered EEG or MRI as clinically indicated. When the child had been evaluated externally, discharge summaries and neuro-imaging reports were acquired; the episode was accepted only if the discharge code corresponded to ICD-10 G40.- or R56.8. Only seizures corroborated by Electronic Medical Record documentation, video footage, or written reports were adjudicated as verified recurrences and included in the survival analysis; episodes documented solely by parental recall without objective evidence were censored at the last confirmed contact date.

### 2.5 Statistical analysis

Analyses were performed using R version 4.3.0 (R Foundation for Statistical Computing, Vienna, Austria). Continuous variables with skewed distributions are presented as median [interquartile range (IQR)]; categorical variables as *n* (%). Between-group comparisons used the Mann–Whitney U test for continuous variables and the Pearson *χ*^2^ or Fisher exact test for categorical variables as appropriate. Risk factors for acute-phase recurrence were first examined in univariable logistic regression models, and then multivariable model; a backward-stepwise selection (*α*-stay = 0.10) was applied to reach the most parsimonious model. Multicollinearity was excluded (all variance-inflation factors < 2.5). Results are expressed as adjusted odds ratios (ORs) with 95% confidence intervals (CIs). The association between acute-phase recurrence and long-term seizure risk was assessed using Cox proportional-hazards regression; proportional-hazards assumption was verified by Schoenfeld residuals (global *p* ≥ 0.11). Potential confounders with *p* < 0.10 in univariable analyses were included in the multivariable model. Kaplan–Meier survival curves were constructed, and differences between groups were evaluated with the log-rank test. Hazard ratios (HRs) with 95% CIs are reported. No missing data were encountered; complete-case analysis was therefore performed. All statistical tests were two-tailed, and *p* < 0.05 was considered statistically significant.

## 3 Results

### 3.1 Baseline characteristics of study participants

**Figure 1:**
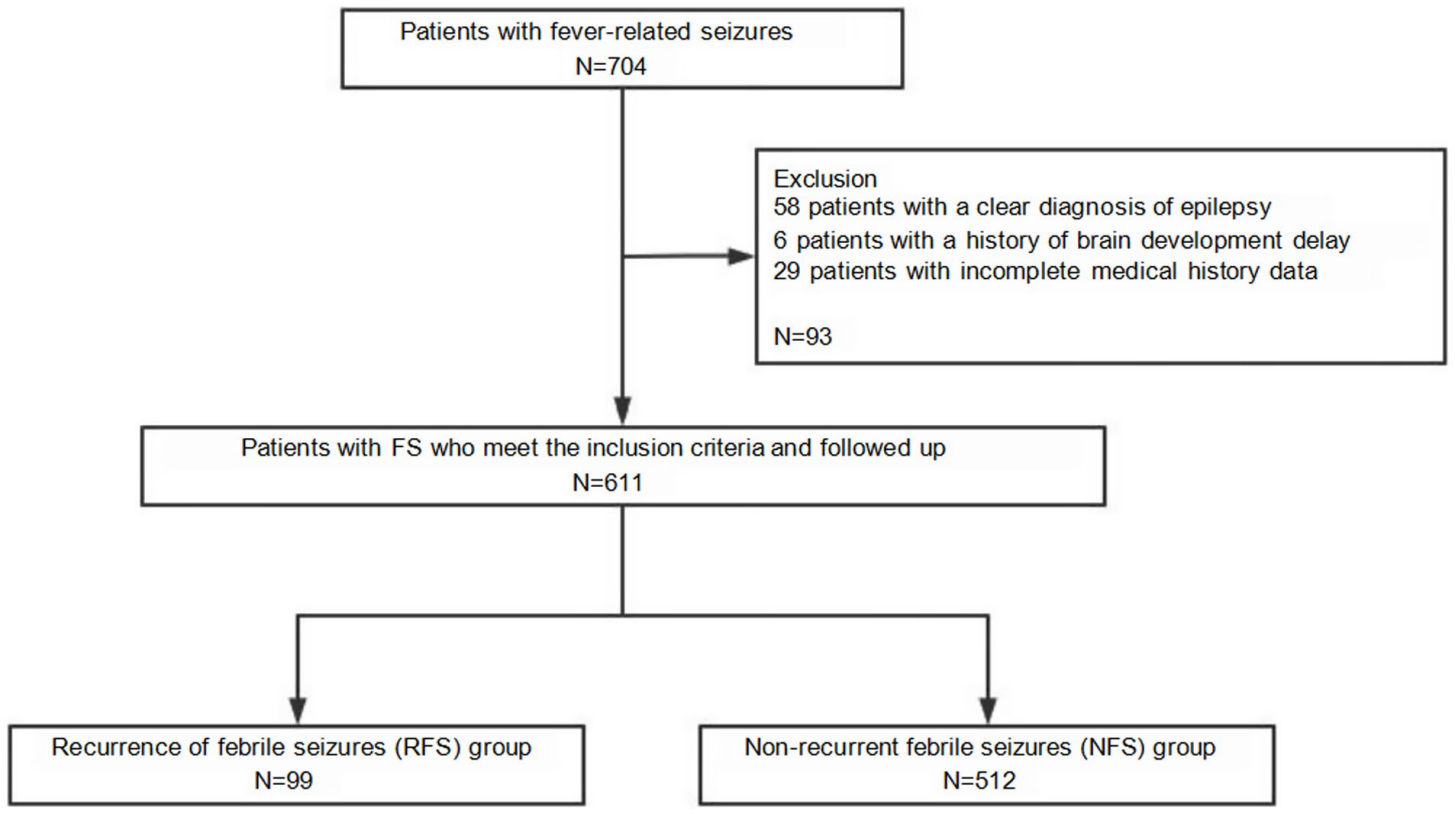
Study enrollment.

A total of 704 pediatric patients presenting with fever and convulsions were enrolled in this study. After rigorous application of exclusion criteria, 93 patients were ultimately excluded from the analysis for the following reasons: 58 were diagnosed with epilepsy, 6 had a history of developmental delay, and 29 had incomplete clinical medical records. Consequently, 611 patients who met the inclusion criteria were included in the analytical cohort (Figure 1). Among them, 414 (67.76%) were male and 197 (32.24%) were female, with a median age of 23 months (16–27 months).

### 3.2 Acute-phase recurrence of FS

**Figure 2:**
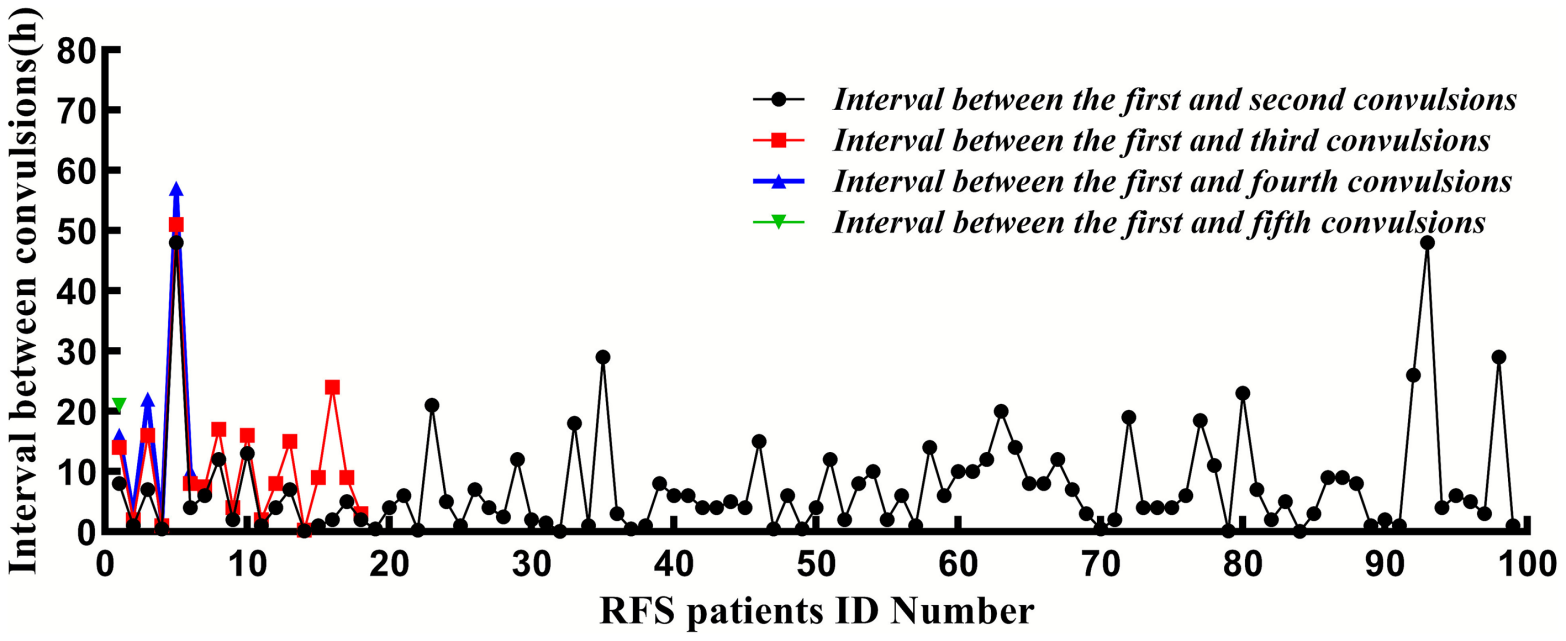
The interval between the convulsion and the first convulsion.

All 611 patients were followed up for 1 week after their first FS. During this period, 399 (65.3%) exhibited simple FS, whereas 212 (34.70%) had complex FS; the latter phenotype accounted for the entire PICU cohort (14/14, 100%) and was markedly over-represented among influenza A-positive cases (71/124, 70.30%). Overall, 99 patients (16.20%) experienced seizure recurrence during the acute phase, while 512 (83.80%) did not. The median time to recurrence was 6 h (IQR: 2.00–11.75 h) after the initial seizure, and 94.95% of recurrent events occurred within 24 h. The distribution of specific recurrence patterns was as follows: 81 patients (13.26%) had 1 recurrences (median interval: 5 h, IQR: 2.00–9.00 h); 12 patients (1.96%) had 2 recurrences (median interval: 8.50 h, IQR: 2.75–16.00 h); 5 patients (0.81%) had 3 recurrences (median interval: 13 h, IQR: 2.63–30.75 h); and 1 patient (0.16%) had 4 recurrences (at 8, 14, 16, and 21 h after the initial seizure). The baseline characteristics of the RFS and NRFS groups are summarized in Table 1. The recurrence intervals during the same fever episode are illustrated in Figure 2.

**Table 1:** Baseline characteristics of study patients.

Variables	Total (*n* = 611)	RFS (*n* = 99)	NRFS (*n* = 512)	Statistic	*p*
Male, *n* (%)	414 (67.76)	67 (67.68)	347 (67.77)	*χ*^2^ = 0.00	0.985
Age (m), M (Q₁, Q₃)	23.00 (16.00, 27.00)	22.00 (16.00, 26.50)	24.00 (17.00, 27.00)	*Z* = −0.60	0.548
Body temperature at FSs (°C), M (Q₁, Q₃)	39.50 (39.00, 40.00)	39.50 (39.00, 40.00)	39.50 (39.00, 40.00)	Z = −1.08	0.281
Body temperature at FSs (°C) *n* (%)				*χ*^2^ = 0.00	0.999
≤39 °C	432 (70.70)	70 (70.71)	362 (70.70)		
>39 °C	179 (29.30)	29 (29.29)	150 (29.30)		
Duration of FSs, M (Q₁, Q₃)	2.00 (1.00, 4.00)	2.00 (1.00, 3.00)	2.00 (1.00, 5.00)	*Z* = −2.51	**0.012**
History of FS, *n* (%)	215 (35.19)	53 (53.54)	162 (31.64)	*χ*^2^ = 17.44	**<0.001**
Family history of FS, *n* (%)	129 (21.11)	23 (23.23)	106 (20.70)	*χ*^2^ = 0.32	0.572
Time from onset of fever to FS, *n* (%)				–	0.204
≤24 h	508 (83.14)	89 (89.90)	419 (81.84)		
24–48 h	87 (14.24)	8 (8.08)	79 (15.43)		
48–72 h	12 (1.96)	2 (2.02)	10 (1.95)		
>72 h	4 (0.65)	0 (0.00)	4 (0.78)		
Complex FS	212 (34.70)	99 (100.00)	113 (22.07)	*χ*^2^ = 222.35	<0.001
Infected site, *n* (%)				–	0.298
Upper respiratory tract infection	516 (84.45)	85 (85.86)	431 (84.18)		
Lower respiratory tract infection	60 (9.82)	12 (12.12)	48 (9.38)		
Gastrointestinal infection	34 (5.56)	2 (2.02)	32 (6.25)		
Urinary tract infection	1 (0.16)	0 (0.00)	1 (0.20)		
Influenza A infection, *n* (%)	101 (16.53)	33 (33.33)	68 (13.28)	*χ*^2^ = 24.18	**<0.001**
Antiseizure medication treatment, *n* (%)	72 (11.78)	33(33.33)	39 (7.62)	*χ*^2^ = 52.78	**<0.001**
Long-term seizure recurrence	120 (19.64)	22 (22.22)	98 (19.14)	*χ*^2^ = 0.50	0.480
Epilepsy, *n* (%)	12 (1.96)	4 (4.04)	8 (1.56)	*χ*^2^ = 1.52	0.218

RFS, recurrence of febrile seizures; NRFS, non-recurrent febrile seizures; M: Z: Mann–Whitney test, *χ*^2^: Chi-square test, −: Fisher exact.

Median, Q₁: 1st Quartile, Q₃: 3st Quartile.

Bold values indicate statistical significance.

### 3.3 Risk factors for acute-phase seizure recurrence

#### 3.3.1 Univariate analysis

Univariate analysis revealed that the proportions of prior FS history and positive for influenza A virus were significantly higher in the RFS group than in the NRFS group (*p* < 0.05). No significant differences were observed between the two groups in terms of sex, age at presentation, body temperature at seizure onset, family history of FSs, or the interval between fever onset and seizure occurrence (*p* > 0.05).

#### 3.3.2 Multivariate logistic regression analysis

**Figure 3:**
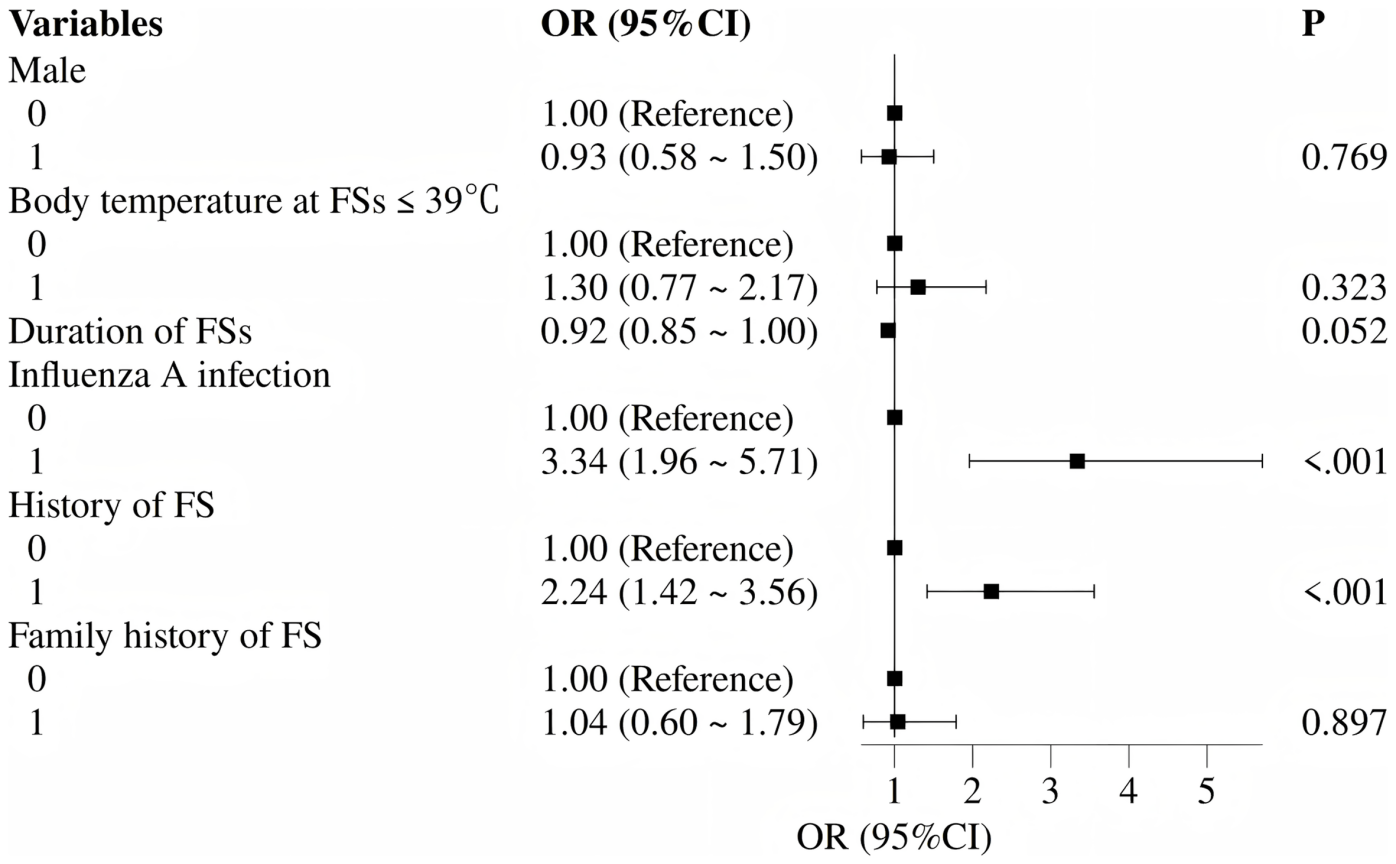
Forest plot of independent risk factors for acute-phase recurrent febrile seizures (multivariate logistic regression).

Based on univariate findings and previously reported risk factors for FS recurrence, six variables were selected for multivariate logistic regression analysis: prior FS history, positive for influenza A, male sex, family history of FSs, body temperature ≤39 °C at seizure onset, and seizure duration. The multivariate analysis identified two independent risk factors significantly associated with acute-phase FS recurrence: prior FS history (OR 2.24, 95% CI 1.42–3.56) and positive for influenza A (OR 3.34, 95% CI 1.96–5.71) (both *p* < 0.05). Detailed results are presented in Table 2 and Figure 3.

**Table 2:** Multivariate logistic regression analyses of the risk factors for acute-phase recurrence of FS.

Variables	Multivariate				
	β	S.E	Z	P	OR (95%CI)
Male	−0.07	0.24	−0.29	0.769	0.93 (0.58 ~ 1.50)
Body temperature at FSs ≤ 39 °C	0.26	0.26	0.99	0.323	1.30 (0.77 ~ 2.17)
Duration of FSs	−0.08	0.04	−1.94	0.052	0.92 (0.85 ~ 1.00)
Influenza A infection	1.21	0.27	4.42	**<0.001**	3.34 (1.96 ~ 5.71)
History of FS	0.81	0.23	3.44	**<0.001**	2.24 (1.42 ~ 3.56)
Family history of FS	0.04	0.28	0.13	0.897	1.04 (0.60 ~ 1.79)

Z: Mann–Whitney test, Bold values indicate statistical significance.

### 3.4 Diagnostic evaluation and outcomes of hospitalized patients with FS

Among the 611 FS patients, 86 (14.08%) required hospitalization, including 14 (2.29%) admitted to the pediatric intensive care unit and 72 (11.79%) treated in general wards (Table 3). Notably, the proportion of influenza A-positive cases was higher among hospitalized patients in both the RFS and NRFS groups (59.38 and 37.04%, respectively). Diagnostic test results were as follows: lumbar puncture performed in 19 (3.33%) patients showed normal cerebrospinal fluid findings; brain MRI was conducted in 56 (9.17%) patients, all of which yielded normal results; and EEG was performed in 47 (7.69%) patients, with only 2 cases (1 in the RFS group and 1 in the NRFS group) showing abnormal findings (poor background activity and increased slow-wave activity).

**Table 3:** Characteristics of admission patients.

Variables	Total (*n* = 611)	RFS (*n* = 99)	NRFS (*n* = 512)	*p*
Out-of-hospital treatment	525 (85.92)	67 (67.68)	458 (89.45)	**<0.001**
In-hospital treatment	86 (14.08)	32 (32.32)	54 (10.55)	
Pediatric intensive care unit	14 (2.29)	10 (10.10)	4 (0.78)	0.004
Neurological ward	72 (11.79)	22 (22.22)	50 (9.77)	
Brain MRI with pathological findings	0/56 (0.00)	0/22 (0.00)	0/34 (0.00)	
EEG with pathological findings	2/47 (4.26)	1/23 (4.35)	1/24 (4.17)	
CSF with pathological findings	0/19 (0.00)	0/14 (0.00)	0/5 (0.00)	
first-ever convulsive seizures	55/86 (63.95)	19/32 (59.38)	36/54 (66.67)	0.496
Seizure lasting >5 min	23/86 (26.74)	8/32 (25.00)	15/54 (27.78)	0.465
Pneumonia	22/86 (25.58)	6/32 (18.75)	16/54 (29.63)	0.264
Influenza A infection, *n* (%)	39/86 (45.35)	19/32 (59.38)	20/54 (37.04)	0.044

RFS, recurrence of febrile seizures; NRFS, non-recurrent febrile seizures; MRI, magnetic resonance imaging; EEG, Electroencephalogram; CSF, cerebrospinal fluid.

Bold values indicate statistical significance.

Table 4 summarises the clinical characteristics and complications of the 14 children admitted to the paediatric intensive care unit (PICU). Median age was 23.50 months (IQR 13.00–25.00), and nine (64.29%) were male. All cases met criteria for complex FS: 8 had status epilepticus and 10 experienced ≥2 recurrent seizures during the acute febrile phase. Influenza A was detected in 11/14 (78.57%). No in-hospital complications or deaths occurred; however, four children (28.57%) subsequently developed epilepsy during follow-up. These findings indicate that PICU admission was prompted by prolonged or frequently recurring seizures rather than by newly identified structural or metabolic pathology.

**Table 4:** Clinical characteristics and complications of children admitted to the pediatric intensive care unit.

Variables	Total (*n* = 14)	RFS (*n* = 10)	NRFS (*n* = 4)	Statistic	*p*
Male, *n* (%)	9 (64.29)	7 (70.00)	2 (50.00)	–	0.580
Age (m), M (Q₁, Q₃)	23.50 (13.00, 25.00)	24.00 (18.00, 25.00)	13.00 (13.00, 13.00)	Z = −0.66	0.508
Body temperature at FSs (°C), M (Q₁, Q₃)	40.00 (39.62, 40.20)	39.90 (39.62, 40.18)	40.15 (39.90, 40.47)	Z = −0.85	0.394
Body temperature at FSs (°C) *n* (%)					1.000
≤39 °C	1 (7.14)	1 (10.00)	0 (0.00)	–	
>39 °C	13 (92.86)	9 (90.00)	4 (100.00)		
Duration of FSs, M (Q₁, Q₃)	2.00 (1.00, 9.12)	1.00 (1.00, 2.00)	10.00 (9.12, 11.25)	Z = −2.25	0.025
Number of seizures during the acute febrile illness period, *n* (%)				–	0.003
1	4 (28.57)	0 (0.00)	4 (100.00)		
2	4 (28.57)	4 (40.00)	0 (0.00)		
3	3 (21.43)	3 (30.00)	0 (0.00)		
4	2 (14.29)	2 (20.00)	0 (0.00)		
5	1 (7.14)	1 (10.00)	0 (0.00)		
History of FS, *n* (%)	7 (50.00)	5 (50.00)	2 (50.00)	–	1.000
Family history of FS, *n* (%)	1 (7.14)	1 (10.00)	0 (0.00)	–	1.000
Time from onset of fever to FS, *n* (%)				–	1.000
≤24 h	11 (78.57)	8 (80.00)	3 (75.00)		
24–48 h	3 (21.43)	2 (20.00)	1 (25.00)		
Infected site, *n* (%)				–	1.000
Upper respiratory tract infection	2 (14.29)	2 (20.00)	0 (0.00)		
Lower respiratory tract infection	11 (78.57)	7 (70.00)	4 (100.00)		
Gastrointestinal infection	1 (7.14)	1 (10.00)	0 (0.00)		
Influenza A infection, *n* (%)	11 (78.57)	7 (70.00)	4 (100.00)	–	0.505
long-term seizure recurrence	5 (41.67)	4 (50.00)	1 (25.00)	–	0.576
Epilepsy, *n* (%)	4 (28.57)	3 (30.00)	1 (25.00)	–	1.000

RFS, recurrence of febrile seizures; NRFS, non-recurrent febrile seizures; M: Z: Mann–Whitney test, *χ*^2^, Chi-square test; −, Fisher exact.

Median, Q₁: 1st Quartile, Q₃: 3st Quartile.

Bold values indicate statistical significance.

### 3.5 Long-term recurrence of FSs and associated factors

Among the 611 children, all completed the final scheduled visit or telephone/WeChat interviews. Individual follow-up ranged from 23 to 48 months, and the median for the entire cohort was 39 months (IQR 37–41). During follow-up, 72 patients received antiseizure medication therapy, including 33 (33.33%) in the RFS group and 39 (7.62%) in the NRFS group (*p* < 0.05). A total of 120 patients (19.64%) experienced seizure recurrence: 22 (22.22%) in the RFS group (median recurrence time: 37 months, range: 24–40 months), including 8 who relapsed during medication, 13 (13.13%) with a single recurrence, and 4 (4.04%) diagnosed with epilepsy; and 98 (19.14%) in the NRFS group (median recurrence time: 38 months, range: 24–41 months), including 7 who relapsed during medication, 75 (14.65%) with a single recurrence, and 8 (1.56%) diagnosed with epilepsy.

**Figure 4:**
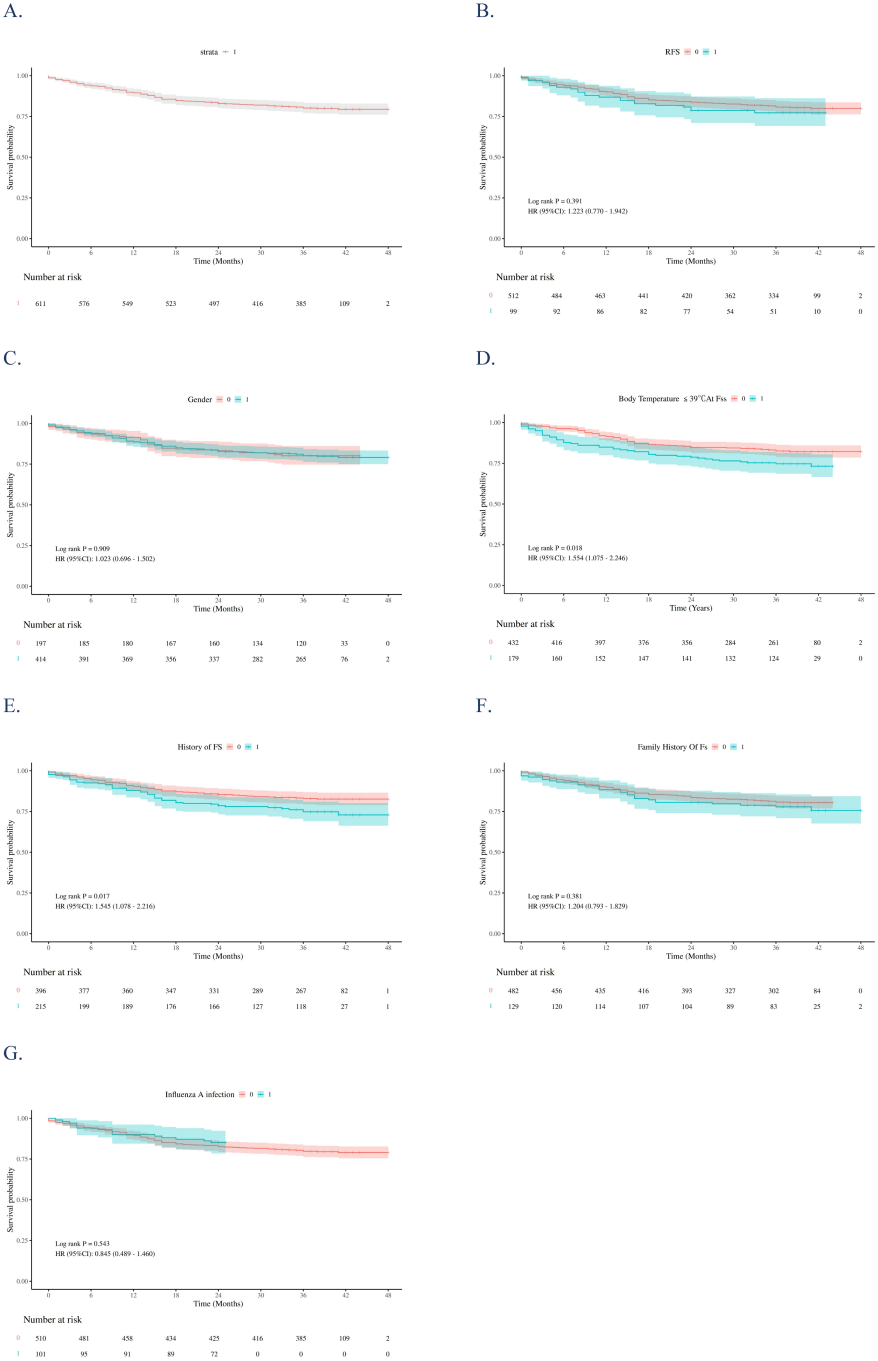
Kaplan–Meier (KM) survival curves for the overall study sample **(A)** and stratifies them by: RFS **(B)**, sex **(C)**, Body temperature at FSs ≤ 39 °C **(D)**, history of FS **(E)**, family history of FS **(F)**, and influenza infection **(G)**. The *p*-values were estimated using the Log-rank test to compare the survival curves of independent groups.

Figure 4 presents Kaplan–Meier survival curves for the entire cohort and stratified by covariates. Compared with the NRFS group, the RFS group showed a slightly higher cumulative recurrence rate, but the difference was not statistically significant (*χ*^2^ = 0.74, *p* = 0.391; HR 1.223, 95% CI 0.770–1.942; Figure 4B). Gender, family history of FS, and influenza infection had no significant impact on recurrence risk (*p* > 0.05; Figures 4C,F,G). In contrast, prior FS history (*p* = 0.017; Figure 4E) and body temperature ≤39 °C at seizure onset (*p* = 0.018; Figure 4D) were significantly associated with higher recurrence risk.

**Figure 5:**
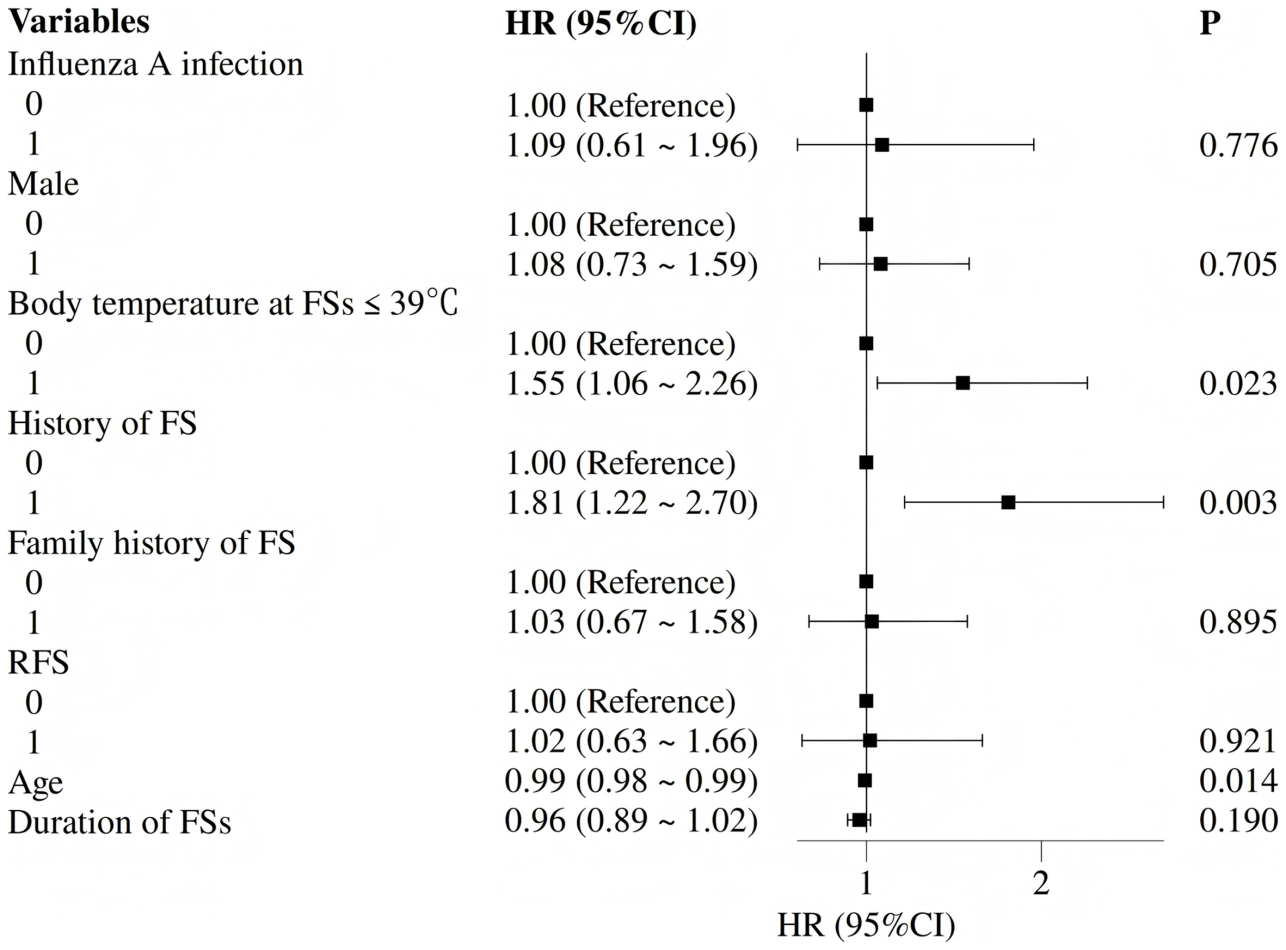
Forest plot of independent predictors of long-term seizure recurrence (multivariate Cox regression).

Cox regression analysis identified predictors of long-term seizure recurrence. Univariate analysis showed that body temperature ≤39 °C (HR 1.55, 95% CI 1.08–2.25), prior FS history (HR 1.55, 95% CI 1.08–2.22), and younger age at onset (HR 0.99, 95% CI 0.98–1.00) were associated with recurrence at the *p* < 0.10 level. After multivariate adjustment, the independent predictors of long-term recurrence were: body temperature ≤39 °C (adjusted HR 1.55, 95% CI 1.06–2.26; *p* = 0.023), prior FS history (adjusted HR 1.81, 95% CI 1.22–2.70; *p* = 0.003), and younger age at onset (adjusted HR 0.99, 95% CI 0.98–0.99; *p* = 0.014) (Table 5 and Figure 5).

**Table 5:** Multivariate Cox proportional hazards analyses of the risk factors for long-term FS recurrence.

Variables	Multivariate				
	*β*	S.E	Z	*p*	HR (95%CI)
Influenza A infection	0.09	0.30	0.28	0.776	1.09 (0.61 ~ 1.96)
Male	0.07	0.20	0.38	0.705	1.08 (0.73 ~ 1.59)
Body temperature at FSs ≤ 39 °C	0.44	0.19	2.27	**0.023**	1.55 (1.06 ~ 2.26)
History of FS	0.59	0.20	2.93	**0.003**	1.81 (1.22 ~ 2.70)
Family history of FS	0.03	0.22	0.13	0.895	1.03 (0.67 ~ 1.58)
RFS	0.02	0.25	0.10	0.921	1.02 (0.63 ~ 1.66)
Age (m)	−0.01	0.01	−2.45	**0.014**	0.99 (0.98 ~ 0.99)
Duration of FSs	−0.05	0.03	−1.31	0.190	0.96 (0.89 ~ 1.02)

Z: Mann–Whitney test, Bold values indicate statistical significance.

## 4 Discussion

This retrospective cohort study with prospective follow-up of 611 children hospitalized for FS systematically evaluated the characteristics of acute-phase recurrence, associated risk factors, current inpatient management practices, as well as long-term recurrence trends and predictive factors. The findings aim to provide evidence-based guidance for identifying high-risk pediatric patients and optimizing clinical management strategies.

### 4.1 Incidence and temporal characteristics of acute-phase recurrence

In our cohort, the acute-phase recurrence rate within 1 week post-initial FS was 16.20% (99/611), with 94.95% of these recurrences occurring within the first 24 h. The median time to recurrence was 6 h (interquartile range: 2.00–11.75 h), and the majority of affected children (81.82%, 81/99) experienced only a single recurrence event. These results are largely consistent with previous literature reporting that “acute-phase recurrences of FS predominantly occur within 24 h of the initial episode and typically manifest as single events” (4, 14, 22), thereby further validating the temporally clustered nature of acute-phase FS recurrences. The notably high recurrence rate within this brief timeframe underscores the clinical imperative for close monitoring of patients in the immediate 24-h period following their first FS, enabling timely identification and management of subsequent seizure events.

### 4.2 Independent risk factors for acute-phase recurrence: history of FS and influenza A infection

Multivariate logistic regression analysis identified two independent risk factors: a prior history of FS (OR 2.24, 95% CI 1.42–3.56) and positive Influenza A infection (OR 3.34, 95% CI 1.96–5.71) (both *p* < 0.05). The association between prior FS history and increased recurrence risk has been extensively documented in previous studies (15, 23–25). This relationship fundamentally reflects either a lower seizure threshold in the child’s nervous system in response to fever stimuli or the presence of underlying epileptiform discharge predisposition. The robust association between influenza A infection and seizure recurrence (OR > 3) constitutes a distinctive finding of the present study. Whether this association arises from rapid temperature fluctuations, systemic inflammation, or residual confounding cannot be elucidated from retrospective data. These findings indicate that pediatric patients with Influenza A infection presenting with fever should be closely monitored for both the occurrence and short-term recurrence of FS. Clinical management protocols should emphasize enhanced temperature surveillance and early therapeutic intervention for these high-risk cases. Pathogen detection was guided by clinical indications rather than a standardized comprehensive panel. Thus, additional infectious causes of fever—such as human herpesvirus 6, adenovirus, respiratory syncytial virus, parainfluenza viruses, or bacterial co-infections—were not systematically assessed beyond the rapid influenza antigen assay. Accordingly, influenza A positivity should be viewed as an associated factor rather than the definitive trigger of recurrence. This limitation is further acknowledged in the Limitations section.

### 4.3 Hospitalization patterns and safety of ancillary tests

Although the overall hospitalization rate was relatively low (14.08%, 86/611), the proportion of Influenza A-positive patients among hospitalized children was significantly higher than that in non-hospitalized patients (59.38% in the RFS group vs. 37.04% in the NRFS group), suggesting a potential association between Influenza A infection and increased disease severity as well as greater need for hospitalization. Only 14 children (2.29%) required admission to the pediatric intensive care unit, indicating that the vast majority of FS cases were mild and did not necessitate intensive care intervention. Nevertheless, every PICU case exhibited complex features—half presented with status epilepticus and nearly 80% had influenza A—highlighting the need for prompt antipyretic and antiviral measures in this small but high-risk subgroup to forestall prolonged seizures and later epilepsy. Regarding ancillary examinations, lumbar puncture (CSF analysis) was performed in 19 patients, cranial MRI in 56, and EEG in 47. Most results were normal, with only two EEG abnormalities (one each in the RFS and NRFS groups) showing poor background activity and increased slow waves. These findings align with previous studies reporting low diagnostic yield of routine neuroimaging and CSF tests in children with FS (26, 27). Therefore, excessive testing may impose unnecessary medical burdens and potential risks unless high-risk indicators — such as focal neurological signs, persistent altered consciousness, or evidence of CSF infection — are present. Complementing this view, Ferretti et al. (19) distilled these into three red-flag categories: predictors of early recurrence, markers of later epilepsy, and indicators of life-threatening differentials (e.g., meningeal signs, prolonged altered consciousness). Integrating such red flags with virological data, as in the present study, should refine future risk-stratification and family counselling.

### 4.4 Long-term recurrence trends and independent predictors

With a median follow-up of 39 months (IQR: 37–41), the long-term seizure recurrence rate in the entire cohort was 19.64% (120/611). Antiseizure medication use differed markedly between groups (33.33% in RFS vs. 7.62% in the NRFS). Consistent with 2011 AAP criteria (2)—which prioritises parental education and fever control and reserves intermittent short-course therapy for situations of severe caregiver anxiety—the high medication prevalence in the acute-recurrence group reflects clinician-initiated rescue prescriptions (usually benzodiazepines or brief levetiracetam) for prolonged/frequent seizures and parental distress, rather than a protocol-mandated maintenance regimen. Importantly, this differential use did not translate into a statistically significant reduction in long-term seizure recurrence (22.22% vs. 19.14%, *p* = 0.480), indicating that any residual protective effect beyond the acute phase is negligible. These findings suggest that while acute-phase recurrence does not directly impact long-term outcomes, it may serve as a potential “early warning signal” for long-term risk (28, 29). With only 12 children developing epilepsy during follow-up, statistical power for epilepsy-specific end-points is limited; therefore, conclusions regarding epilepsy incidence remain exploratory and require validation in larger, multi-centre cohorts.

In identifying independent predictors of long-term FS recurrence through Cox regression analysis, we found that a peak temperature ≤ 39 °C, a history of prior FS, and younger age at onset were significant predictive factors (all *p* < 0.05). These conclusions align with multiple studies that have established an association between “lower seizure temperature” (≤39 °C) and increased risk of FS recurrence (10, 12, 14). Children whose seizures can be triggered by relatively low body temperatures may possess a “low-threshold” characteristic in their nervous system, where neuronal excitability crosses the discharge threshold even with minimal fever elevation. This finding underscores the clinical importance of enhanced long-term follow-up for patients who experience seizures at temperatures ≤39 °C, with particular attention to the potential for recurrence. A history of prior FS emerged as a classic predictive factor (30, 31). This result emphasizes the fundamental role of comprehensive medical history-taking, with specific focus on prior seizure episodes, in risk assessment. Furthermore, younger age at onset was also identified as a significant predictor of FS recurrence. Literature indicates that an initial FS occurring before 14–16 months of age represents one of the strongest predictors of recurrence (32). Consistent with these findings, our research further validates the critical importance of age as a factor in recurrence risk assessment. Therefore, for FS patients with an early age of onset, clinicians should implement closer monitoring of their neurodevelopmental trajectory and ensure timely intervention during febrile episodes.

FS exhibits a clear familial aggregation, typically following polygenic or autosomal-dominant patterns with incomplete penetrance. Multiple genes and chromosomal abnormalities have been reported to be associated with FS (33, 34). Because genetic analyses were not available in this retrospective cohort, future prospective studies should include targeted or panel sequencing to integrate these monogenic risk factors into comprehensive risk-stratification models and to delineate precise genotype–phenotype relationships.

Equally important, clinicians should supplement medical follow-up with structured caregiver education on seizure recognition and first-aid management. Loussouarn et al. (35) demonstrated that this intervention significantly reduces parental post-traumatic stress, sleep disruption, maladaptive parenting practices, and the fear-driven overuse of potentially harmful antipyretics, while also curbing unnecessary emergency visits. Integrating such standardized educational sessions into routine care, therefore improves both family outcomes and health-service efficiency.

## 5 Significance and limitations of the study

The primary contributions of this study are as follows: First, it elucidated the temporal patterns of acute-phase recurrence and identified two strongly associated independent risk factors — prior FS history and positive Influenza A infection — providing precise targets for early clinical warning. Second, it revealed core predictors of long-term recurrence [seizures triggered by low-grade fever (temperature ≤39 °C), prior FS history, and young age at onset], facilitating individualized risk assessment and personalized follow-up planning. Third, it demonstrated the low diagnostic yield of routine ancillary tests (e.g., cranial MRI and cerebrospinal fluid analysis), lending empirical support to the principle of “avoiding overtreatment.”

However, the study has several limitations. As a retrospective cohort, this study is susceptible to selection and information bias, missing data, and unmeasured confounding. Consequently, the observed associations (e.g., influenza A and prior FS history) should be viewed as hypothesis-generating rather than causal. Prospective, protocol-driven investigations are required to confirm these risk estimates and to establish true cause-effect relationships. Furthermore, as a single-center investigation, the geographical representativeness of the sample may be limited. Additionally, Influenza A positivity was determined solely through clinical testing (e.g., throat swabs) without distinguishing viral load levels or specific subtypes (e.g., H1N1, H3N2). The lack of comprehensive etiologic work-up for all febrile episodes may have led to residual confounding by unmeasured infections. Future multiplex PCR panels incorporating influenza B and additional respiratory viruses are needed to quantify the specificity of the influenza A signal and to determine whether other pathogens confer similar short-term seizure risk. Besides, long-term follow-up data relied primarily on parental reports, which may have led to underreporting of mild recurrence events, despite mitigation through scheduled follow-ups. In addition, the low number of PICU admissions precluded multivariable modelling of severity predictors; establishing a multicentre severe-FS registry would overcome this constraint and refine risk stratification. Then, the absence of genetic data precluded evaluation of monogenic susceptibility (e.g., SCN1A, PCDH19) that may amplify both acute-phase recurrence and long-term epilepsy risk; prospective studies should integrate targeted or exome sequencing into comprehensive risk models. Lastly, because neuroimaging and CSF examinations were restricted to predefined high-risk presentations, the observed low yield may not reflect the true prevalence of abnormalities in an unselected FS population; this potential selection bias limits generalisability and warrants interpretation with caution.

## 6 Conclusion

In summary, this study confirmed that the acute-phase recurrence rate of FS in children is 16.20% (with key risk factors being prior FS history and Influenza A infection), while the long-term recurrence rate is 19.64%. Independent predictors of long-term recurrence include fever ≤39 °C, prior FS history, and young age at onset. Clinically, heightened attention should be paid to high-risk children exhibiting these factors, with optimization of both acute-phase monitoring (particularly within the first 24 h) and long-term follow-up strategies. Unnecessary ancillary testing should be avoided to achieve precise, individualized management goals, ultimately improving patient outcomes and reducing healthcare resource waste. Finally, routine structured caregiver education decreases parental anxiety and curbs fever-phobic overuse of antipyretics, thereby improving family-centred outcomes.

## Data Availability

The raw data supporting the conclusions of this article will be made available by the authors, without undue reservation.
